# Research on rapid construction methods and evaluation of health education resources in public health emergencies based on knowledge development

**DOI:** 10.3389/fpubh.2025.1686843

**Published:** 2025-12-17

**Authors:** Rong Huang, Yi Zou, Lifeng Zhou, Tao Jiang

**Affiliations:** 1 School of Humanities, Zhuhai City Polytechnic, Zhuhai, China; 2 School of Humanities and Management, Guilin Medical University, Guilin, China; 3 Huinan Community Health Service Center, Shanghai, China

**Keywords:** epidemic bulletin, health education resources, knowledge graph, library and information science, public health emergency, technology acceptance model

## Abstract

**Objectives:**

This study aims to construct and validate an interdisciplinary framework based on Library and Information Science (LIS) to improve the timeliness and accuracy of health education resource development during public health emergencies, and to provide a practical technical approach and theoretical framework through a complete “analysis-generation-evaluation” cycle for resolving the conflict between “information overload” and “precise targeting” in crisis communication.

**Methods:**

A total of 1,026 epidemic bulletins from various levels of government in China (2020–2024) were collected as the primary data source. In-depth knowledge development was achieved through core Library and Information Science (LIS) methods such as knowledge graph construction, thematic analysis, natural language processing (NLP), and association rule mining. Building upon these analytical results, an automated resource generation system was developed based on the Technology Acceptance Model (TAM). The system was subsequently evaluated using questionnaires administered to 305 users.

**Results:**

A topic modeling analysis was conducted on 1,026 epidemic announcements, revealing five themes, with preventive measures being the most prominent (32.7%). Association rule mining indicated significant co-occurrence patterns among key protective factors (support >0.6, confidence >0.8). An automated resource generation system based on the Technology Acceptance Model (TAM) was evaluated using 305 valid questionnaires, showing a high level of user acceptance. Specifically, Path analysis confirmed that perceived usefulness (*β* = 0.42, *p <* 0.001) was the strongest predictor of behavioral intention, followed by perceived ease of use (*β* = 0.31, *p <* 0.01). Logistic regression further showed that trust in official information sources (OR = 2.05) and eHealth literacy levels (OR = 1.87) were important factors influencing perceived resource effectiveness.

**Conclusion:**

The core of this study established a pathway that rapidly and automatically converts authoritative epidemic announcements into personalized health education resources. The framework utilizes LIS technologies such as knowledge graphs and association rule mining to analyze the content of the announcements and achieve automatic resource generation. Empirical research shows that user acceptance of these resources depends primarily on their perceived usefulness and ease of use, with eHealth literacy playing an important moderating role in this process. The study’s “analyze-generate-evaluate” closed-loop model can be extended to other crisis situations.

## 1 Introduction

The persistent threat of public health emergencies poses severe challenges to the global health governance system (1). An effective response largely depends on the precise and rapid dissemination of health education information (2). During the COVID-19 pandemic, timely health education faced numerous challenges, including information overload, the “infodemic” rapidly evolving information, insufficient scientific accuracy, and challenges to the credibility of information sources (3, 4). Traditional health education resource development models, such as the ADDIE model, struggle to meet the dynamically changing public information needs during public health emergencies due to their long development cycles and limited flexibility (5).

In response to these challenges, information technology has opened new pathways for innovation in health education (6). Library and Information Science (LIS) provides a solid theoretical and methodological foundation in areas such as knowledge organization, information retrieval, and user information behavior, offering a promising framework to overcome the limitations in current public health communication (7). Evidence from organizations such as the World Health Organization (WHO) indicates that knowledge-driven technologies can significantly enhance the efficiency and accuracy of health communication (8). Existing research has begun to explore the application of LIS technologies, often treating them as isolated tools for epidemic analysis or confining user evaluation to basic satisfaction surveys (9, 10). However, a significant gap remains: there is a lack of a coherent framework that organically integrates the automated semantic analysis of authoritative texts, the intelligent generation of educational resources, and the validation of user adoption mechanisms (11).

To address this gap, this study, rooted in the field of LIS, proposes a data-driven framework for the automated generation and evaluation of health education resources. It aims to systematically tackle three core issues in emergency health education:

1. “What to generate?” – Guided by information behavior theory, we systematically extract and structure core knowledge granules from authoritative texts using natural language processing (NLP) and knowledge graphs.
2. “How to generate?” – We develop an automated system that reassembles these granules into personalized resources, with formats adapted via a multimodal mechanism to enhance compatibility with different user profiles.
3. “How well is it generated?” – We move beyond simple satisfaction metrics by employing a Technology Acceptance Model (TAM) based evaluation to quantitatively measure the perceived usefulness and ease of use of the generated resources, with eHealth literacy tested as a key moderator, thus empirically validating the “Mindsponge” filtering process.

The study presents a complete “analyze-generate-evaluate” closed-loop framework. This framework introduces information behavior theory to guide content mining, utilizes natural language processing NLP and knowledge graph technologies for automated knowledge restructuring and intelligent adaptation, and establishes an evaluation system based on the Technology Acceptance Model (TAM) to scientifically measure user behavioral intention.

The theoretical rationale for this approach is further supported by the Granular Interaction Thinking Theory (GITT), which provides a micro-level explanation for understanding individual health decision-making mechanisms in public crisis situations (12). GITT posits that individuals’ values, attitudes, and subsequent behaviors are dynamically shaped through continuous, granular interactions with the external information environment, processed via internal psychological filtering mechanisms such as the “Mindsponge” process (13). This theory aligns closely with the core concerns of LIS regarding the interaction between people, information, and technology. It guides the research design through an empirical “information absorption” process: text analysis deconstructs the information environment, resource generation optimizes information “granules” for user compatibility, and effect evaluation quantitatively measures the absorption outcome, with constructs including perceived usefulness (absorption benefits), perceived ease of use (absorption costs), and eHealth literacy as a key moderating variable.

Ultimately, this study provides a feasible technical approach and practical method for shifting health communication from “information broadcasting” to “targeted dialogue” in crisis situations (14–17). By establishing a systematic process from authoritative information sources to personalized educational resources, it demonstrates the application of LIS knowledge development methods and aims to enhance both the quality and efficiency of health education initiatives, thereby contributing to improved public health emergency preparedness and response (18, 19).

## 2 Methods

**Figure 1:**
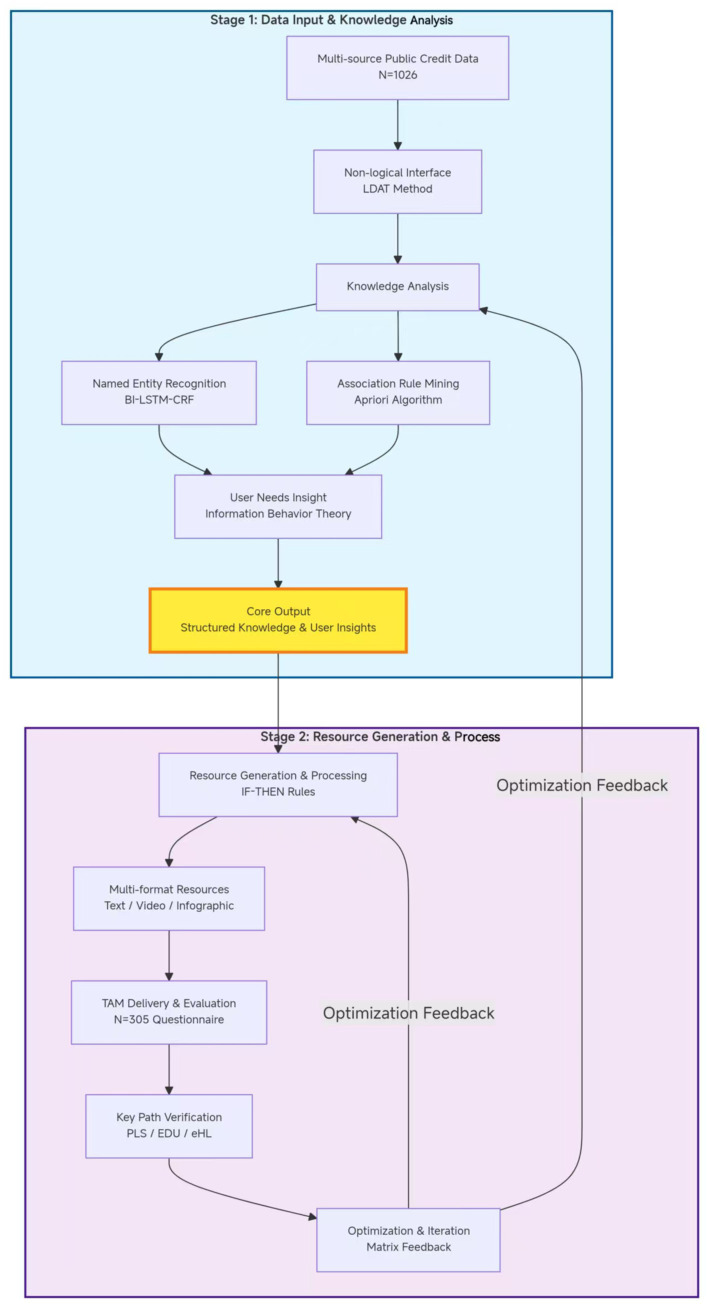
Flowchart for knowledge analysis and resource generation from credit data.

This study adopted a multi-phase mixed-methods research design, encompassing four sequential stages: data collection, knowledge analysis, resource construction, and effectiveness evaluation. It aims to build a complete evidence chain from authoritative text analysis to user effect validation. The core of the approach lies in ensuring that the output of text analysis can directly guide the creation of educational resources and ultimately validate its effectiveness through social data analysis. Both quantitative and qualitative methods from LIS were comprehensively integrated throughout the research process. The overall diagram is as follows Figure 1.

### 2.1 Data sources and preprocessing

#### 2.1.1 Data sources

The research uses epidemic bulletins as the data source. It includes the National Health Commission of China, the provinces of Jiangsu, Hubei, and Guangdong, as well as the health and wellness commissions of their respective municipal-level administrative units. As a cornerstone of public health communication, epidemic bulletins are issued by legally authorized institutions to ensure the reliability of information sources, a principle strongly advocated by the World Health Organization (20) for effective emergency risk communication. In terms of spatial dimensions, the content of these bulletins exhibits significant variations corresponding to different administrative levels (national, provincial, local) and localized epidemic risk levels, reflecting the tiered response system in public health governance (21). In terms of temporal dimensions, the epidemic bulletins are released regularly. But the focus and instructions in epidemic bulletins evolve with the development stage of the epidemic. This dynamic communication strategy aligns with established crisis communication models, which prescribe customized information dissemination for the initial, ongoing, and resolution/normalization phases of a crisis (22). Epidemic bulletins issued by health commissions at all administrative levels in China between January 2020 and December 2024 were collected using a stratified random sampling approach. Based on China’s epidemic prevention and control policies and academic research results, China’s epidemic prevention and control is divided into three stages (23–26). The stratification was conducted first by epidemic phase—initial phase (January to March 2020), sustained phase (April 2020 to November 2022), and normalized prevention phase (December 2022 to December 2024). China’s administrative management system is a typical pyramid structure. To accurately reflect the structure of China’s administrative and epidemic information dissemination system, it is important to ensure that the samples can simultaneously capture the macro-level policy directions at the national level, the meso-level regional strategies at the provincial level, and the micro-level grassroots practices at the city live (27). Sampling by administrative level allocated national-level sources to 15% of the sample, provincial-level to 35%, and municipal-level to 50%. A “unique identifier” was created for each announcement data, that is, an ID in the format of “Date_Province_DataType” is generated. A process of identification, comparison, processing, and recording was adopted to ensure that each announcement data is unique. Based on this design, a corpus of 1,026 official bulletins was constructed, comprising a total of approximately 3.12 million Chinese characters.

#### 2.1.2 Date preprocessing framework

This study employed a systematic text preprocessing framework. It includes two stages: data preprocessing and text analysis. The process began with data cleaning, including the removal of HTML tags, non-Chinese characters, and structured table formats. This was followed by word segmentation using the Language Technology Platform (LTP) toolkit, integrated with a customized medical dictionary covering domain-specific terms such as virus names and symptom descriptions. Next, text analysis was conducted. Text analysis is both a text preprocessing tool and a key methodology that bridges previous and subsequent steps (28). It builds on the clean text obtained from data preprocessing and provides the structured knowledge elements needed for knowledge analysis. It is the core component for achieving the three major research goals of “automated generation,” “scientific reliability,”and “precise targeting” (29, 30). Named entity recognition was performed using the BIO (Beginning-Inside-Outside) tagging scheme to identify seven categories of entities: viruses, symptoms, drugs, preventive measures, institutions, locations, and temporal expressions. A total of 12,450 entities were annotated in the entity recognition phase, with “symptoms” and “viruses” being the most prominent, accounting for 38.5 and 25.1% of all recognized entities, respectively. Finally, denoising was completed by removing common stop words (e.g., de, he, zai) and low-frequency words (i.e., those appearing fewer than five times). After denoising, the total number of text entries was reduced from approximately 1.5 million to about 890,000, −a decrease of around 40%-, while effectively retaining about 15,000 high-frequency core terms.

### 2.2 Knowledge analysis methods

The core objective of this phase is to systematically extract from epidemic announcements the key knowledge elements that can be used to develop health education resources.

#### 2.2.1 Knowledge graph construction

The goal of this stage is to build a dynamic and structured knowledge base for the resource generation system. A knowledge graph of epidemic reports was constructed through the comprehensive use of the Neo4j graph database. The specific workflow is as follows:

1. Entity Extraction: Named entity recognition was performed on bulletin texts using a BiLSTM-CRF model, primarily covering categories such as viruses, symptoms, and preventive measures. The model achieved an F1 score of 0.86.
2. Relation Extraction: A combination of rule-based pattern matching (e.g., “transmission route” patterns) and a BERT-based relation classification model was employed to automatically identify semantic relationships between entities.
3. Knowledge Fusion: Synonymous entities (e.g., “NCP” and “COVID-19”) were merged, and contextual disambiguation was applied to polysemous terms (e.g., “geographical isolation,” which may refer to medical or geographic isolation).
4. Visualization and Query: The knowledge graph supports visualization and interactive retrieval using the Cypher query language (e.g., `MATCH (n: Virus)-[r: TransmissionMode]- > (m) RETURN n,r,m`).

The statistical distribution of node types, relationship categories, and representative examples in the constructed knowledge graph is summarized in Table 1.

**Table 1:** Statistics of knowledge graph node types and relationship categories.

Node type	Count	Relationship type	Count	Example
Virus	24	Transmission pathway	78	Omicron variant → airborne transmission
Symptom	37	Preventive measure	126	Wearing masks → prevention of infection
Preventive measure	58	Therapeutic drug	42	Lianhua Qingwen → alleviation of symptoms
Medical institution	1,150	Geographical location	3,892	Suzhou City → reported cases
Vaccine type	12	Vaccination schedule	18	Inactivated vaccine → requires two doses

#### 2.2.2 Topic modeling and association rule analysis

1. *Latent Dirichlet allocation (LDA) topic modeling:* Topic modeling was conducted using the Latent Dirichlet Allocation (LDA) algorithm. The number of topics was initially set at K = 10 and optimized to K = 5 based on perplexity evaluation. The model was implemented using the Gensim library with the following parameters: *α* = 0.1, *β* = 0.01, and 1,000 iterations.
2. *Association rule mining:* The Apriori algorithm was employed to mine frequent itemsets related to preventive and control measures. The minimum support threshold was set to 0.3, and the minimum confidence threshold was set to 0.7.
3. *Chi-square test:* The Chi-square (*χ*^2^) test was used to examine the statistical association between types of health education resources (e.g., infographic, video, Q&A) and user characteristics, including age and educational attainment.
4. *Binary logistic regression:* A binary logistic regression model was constructed with “perceived effectiveness of health education resources” as the dependent variable (1 = effective, 0 = ineffective). Independent variables included health literacy, preferred information channels, and personal pandemic experience.

#### 2.2.3 Multimodal adaptation mechanism

The multimodal adaptive mechanism plays a core “generator” role in the “analyze-generate-evaluate” framework (31). It inherits the output from knowledge analysis and directly drives the concrete actions of resource generation, serving as an intelligent bridge between “what the content is” and “how the form should be.” This mechanism is implemented through a conditional selection function, with the user profile as input and the recommended multimodal resource format as output. The internal rules of the function are designed based on prior research and theoretical inferences about the information processing characteristics of different user groups.

Resource formats were adapted to user characteristics through a conditional selection mechanism:

1. For older adults: When users are aged 60 or above, the system automatically adopts a combination of “Audio Narration + Large-Font Text.”
2. For low education level groups: When a user’s educational background is marked as “Below High School,” the system intelligently matches them with a “Short Video + Infographic” presentation format.
3. Universal solution: For user groups that do not meet the above characteristics, the default provision is “Interactive Q&A + Long-form Text” for deep cognitive engagement.

### 2.3 Mechanism for health education resource generation

**Figure 2:**
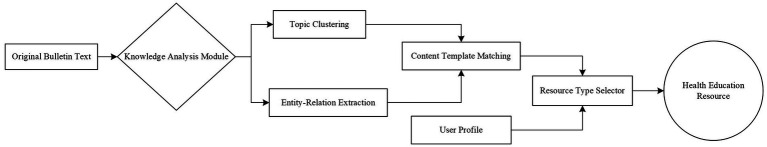
Architecture of the intelligent generation system for health education resources.

This stage is a key step in transforming text analysis results into educational resources that can be accepted by specific user groups. Its design principle is to match the “knowledge elements” obtained from text analysis with the “user characteristics” known through preliminary research or literature. Based on the topics, entities, and association rules output from the aforementioned knowledge analysis stage, an automated framework for generating health education resources has been developed. As shown in Figure 2.

*Examples of Generation Rules:* IF Topic = “Virological Characteristics” AND User Age ≥ 60, THEN adopt a “video + audio narration” format; IF an association rule involving “vaccine efficacy” is detected, THEN insert comparative charts with key data; IF the entity “Omicron” is recognized, THEN link to a dedicated science communication page on virus variants.

### 2.4 Evaluation design based on the technology acceptance model (TAM)

To verify the actual effectiveness of the resources generated by this framework and to answer the question of “how well they are generated,” an evaluation framework was developed based on Davis’s Technology Acceptance Model (TAM) as the theoretical foundation. The core of this framework is binary logistic regression analysis, used to assess users’ acceptance of the generated health education resources.

#### 2.4.1 Regression analysis

This study uses logistic regression analysis, for three main reasons:

1. *Consistency with the research objectives:* One of the goals of this study is to assess the “effectiveness” of generated resources. Logistic regression models are well-suited for handling dependent variables that are binary (such as “effective” vs. “ineffective”), allowing direct prediction of the probability that a resource is deemed effective.
2. *A bridge connecting text analysis and social data:* Regression analysis allows us to incorporate outputs from text analysis alongside social data in the model, thereby quantifying their independent contributions to resource effectiveness.
3. *An empirical basis for “precise deployment”:* By identifying key influencing factors, we can provide a data-driven basis for optimizing the rules in the resource generation knowledge base.

This analysis is rooted in the TAM model, whose intrinsic logic is that users’ acceptance of technology (intention to use resources) is jointly influenced by their perceptions (usefulness, ease of use) and external variables (such as user characteristics and system characteristics).

#### 2.4.2 Definition of variables, measurement methods, and theoretical roles

1 Independent variables: (i.e., technology perception variables), perceived usefulness (PU), perceived ease of use (PEOU).

*Perceived Usefulness (PU).*

Measurement method: In the questionnaire, we used a 5-point Likert scale (1 = strongly disagree, 5 = strongly agree) for measurement.

Theoretical basis: A core construct of TAM. In this study, it measures the “information granules” refined and optimized through text analysis, which directly reflect the quality of the resource content.

*Perceived Ease of Use (PEOU).*

Measurement method: Also measured using a 5-point Likert scale. Representative item: “Learning how to use these resources (such as watching videos, reading infographics) is easy for me.”

Theoretical basis: Another core construct of TAM. It corresponds to the multimodal resource generation mechanism in this study.

2 Dependent variable: (i.e., behavioral outcome variable), using behavioral intention (BI)

Measurement method: Measured through the question in the questionnaire “Do you think this resource is helpful for your health decisions?” Respondents who choose “Agree” or “Strongly agree” on a 5-point Likert scale are coded as 1, otherwise as 0. Theoretical basis: In the TAM, behavioral intention is the ultimate dependent variable of technology acceptance and is the most direct indicator for predicting actual usage behavior.

3 Moderating Variable: User Characteristic VariableElectronic Health Literacy (eHL)

Measurement Method: Measured using the eHealth Literacy Scale (eHEALS), which is widely used in the international academic community. This scale contains 8 items, also rated on a 5-point Likert scale. Participants with total scores above the sample median are coded as the “high eHL” group, while the rest are classified as the “medium/low eHL” group.

Theoretical Basis: This variable is a core operational indicator of the ‘thinking sponge’ filtering ability in the Granular Interactive Thinking Theory (GITT).

4 Demographic Characteristics

Measurement Method: Collected directly in the demographic section of the questionnaire. Age: Treated as a categorical variable, such as “18–30 years,” “31–59 years,” “60 years and above.”Education Level: Treated as a categorical variable, such as “High school or below,” “College/Undergraduate,” “Postgraduate and above.”

Theoretical Basis: These characteristics are the cornerstone of user profiling and serve as the direct basis for multimodal adaptation in the resource generation rule library.

#### 2.4.3 Data collection and model validation

##### 2.4.3.1 Data collection

This study collected data using a stratified sampling method. First, based on the stratification variables of epidemic severity and regional distribution, three representative provinces were selected: Hubei Province (initial epicenter and high-intensity control area), Guangdong Province (southeastern coastal economic center with ongoing importation pressure), and Jiangsu Province (economically strong province on the eastern coast with efficient implementation). Within each province, participants were recruited using a combination of quota sampling (controlling for age and gender) and snowball sampling.

In the survey design, automatically generated educational resources (with their topic composition shown in Table 1 and examples provided in Table 2) were systematically integrated to assess the impact of popular science information on public knowledge and attitudes across different topics. The specific integration method and participant procedure are as follows:

1. *Questionnaire Structure Design and Resource Allocation:* The core part of the questionnaire was designed as a module for topic exposure and measurement. The popular science texts generated in Table 2 were used as key stimulus materials. To ensure the ability to analyze the independent effects of different topic resources, a between-group design was employed: participants were randomly assigned to one of five experimental groups, with each group exposed to all the popular science texts and corresponding questions under a single main topic (e.g., the “Prevention and Control Measures” group).
2. *Participant Involvement Process:*

**Table 2:** Topic modeling identified five major themes and their relative proportions.

Major themes	Relative proportion
Preventive and control measures	32.7%
Virological characteristics	24.1%
Case data	18.5%
Medical resource allocation	15.2%
Science communication	9.5%

*Step One:* Collection of Basic Information and Informed Consent. participants first signed the informed consent form and complete their demographic information.

*Step Two:* Exposure to Subject Resources. Then, based on their randomly assigned group, the system presents participants with a set of popular science texts on specific topics (for example, participants in the “Virological Characteristics” group will read texts about topics such as “Aerosol Transmission of Omicron”). Participants were required to read these materials carefully.

*Step Three:* Immediate Effect Measurement. After reading the resources on the specified topic, participants are required to immediately complete a series of measurement questions directly related to the topic content. These questions use a five-point Likert scale with 35 items and are designed to assess their level of knowledge comprehension, risk perception, and behavioral intention to take protective actions.

*Step Four (Optional):* Cross-Exposure Measurement. To further investigate interactions between topics, after completing the measurement of the main topic, participants in some groups may be additionally exposed to resources for a secondary topic and complete the corresponding measurement.

Through the above design, the automatically generated educational resources were incorporated as the core intervention variable in the survey experiment, enabling accurate tracking the direct impact of information on different topics on participants’ feedback.

A total of 327 participants were recruited and surveyed using a 35-item questionnaire with a 5-point Likert scale. Ultimately, 305 valid questionnaires were obtained, resulting in an effective response rate of 93.3%. The demographic characteristics of the valid sample (N = 305) were as follows: ages ranged from 18 to 76 years (mean = 42.3 years), gender distribution was relatively balanced (48.2% male), and the majority of participants (62.6%) had received higher education.

##### 2.4.3.2 Model validation

1. Data analysis was conducted using SPSS 25.0, applying the following procedures:
2. Reliability and validity testing: Cronbach’s *α* > 0.8; Average Variance Extracted (AVE) > 0.5
3. Structural equation modeling: Path coefficients were assessed using Partial Least Squares Structural Equation Modeling (PLS-SEM)
4. Moderation effect testing: Hierarchical regression analysis was used to examine interaction effects

## 3 Results

### 3.1 Multidimensional knowledge characteristics of government bulletins

An in-depth analysis of the 1,026 epidemic bulletins revealed the structured features and temporal evolution patterns of information dissemination during public health emergencies. Topic modeling identified five major themes and their relative proportions.

The distribution of topics varied significantly across different epidemic phases (*χ*^2^ = 86.32, *p <* 0.001). In the initial phase, the focus was primarily on virological characteristics (40.2%) and case data (30.1%), whereas in the normalized prevention phase, the focus shifted to preventive measures (38.5%) and science communication (15.3%).

*Association rule mining uncovered strong patterns of co-occurrence among key preventive elements. High-frequency binary item sets included:*

- “Mask-wearing and frequent handwashing” (support = 0.78, confidence = 0.93)

This indicates that during the pandemic, “wearing masks and washing hands frequently” was the most fundamental and widely adopted combination of personal protective measures. In the public’s awareness, “wearing masks” and “washing hands frequently” were bundled together as a unified preventive behavior.

- “Nucleic acid testing and health code verification” (support = 0.65, confidence = 0.89)

This indicates that during the pandemic period, “nucleic acid testing and health code verification “was a commonly used combination of social prevention and control measures. The act of “nucleic acid testing” is mainly for the purpose of “health code verification,” reflecting the deep integration of the two measures.

*Notable ternary itemsets included:*

- “Omicron variant – Boostervaccination – breakthrough infection” (support = 0.51, confidence = 0.82)

“Omicron variant – Booster vaccination” association is important because of the connection between viral characteristics and vaccine protection mechanisms. The combination of “Omicron variant-Boostervaccination-Breakthrough Infection” reflects a new trend in health information dissemination in the pandemic era, where “risk communication” acknowledging breakthrough infections replaces unrealistic “absolute safety promises.”

In summary, these patterns reveal the intrinsic interdependencies among control measures. For instance, when bulletins mentioned “crowd gatherings,” 92.3% also emphasized “mask-wearing,” highlighting a consistent public health messaging strategy.

The construction of the epidemic knowledge graph enabled semantic associations among extracted information. The graph contained 1,281 nodes and 4,156 edges, with the “virus–prevention measures” subgraph exhibiting the highest density (0.76). Key path analysis revealed that the most frequently occurring knowledge chain was:

“Omicron → airborne transmission → mask-wearing → 95% protective efficacy” (appearing 1,024 times).

Moreover, the graph exposed gaps in public knowledge. For example, the association strength between “aerosol transmission” and “ventilation measures” was only 0.34, suggesting that this critical information was insufficiently emphasized in official communications.

### 3.2 Automated generation of health education resources

Based on the preceding analyses, a dynamic resource generation system was developed that automatically completes tasks using preset algorithms and rules. Its core mechanism consists of the following components:

#### 3.2.1 Content mapping rule base

Analytical outputs were systematically transformed into structured elements of science-based educational content:

1. *Topic → Content Framework:* For example, the topic “Preventive and Control Measures” (as identified in our topic modeling) was mapped to a three-level content structure, including Personal Protection (a sub-category), Community Management, and Travel Policies.
2. *Entity → Knowledge Card:* When the entity “Lianhua Qingwen” was identified, a corresponding knowledge card was inserted stating: “U*sage:* Symptom relief only; not for prevention.”
3. *Association Rule → Risk Alert:* If co-occurrence of “mass gatherings” and “absence of mask-wearing” was detected (a pattern mined from the “Preventive and Control Measures” topic), a contextualized risk alert case was automatically generated

#### 3.2.2 Knowledge update trigger

The system was designed to automatically detect new entities (e.g., “XE variant”) in bulletins and activate a knowledge update pipeline: Entity recognition → Retrieval of authoritative sources → Generation of knowledge triples → Knowledge graph update → Release of science briefs, as shown in Table 3 (Example of generated health education resources under the theme “Preventive and Control Measures”; Sub-topic: Personal Protection).

**Table 3:** Example of generated health education resources under the theme “preventive and control measures”; sub-topic: personal protection.

Knowledge element source	Original bulletin content	Converted popular science text
Association rule	“Wearing masks should be implemented simultaneously with maintaining social distancing”	Masks can block more than 80% of droplets; however, when the distance is less than 1 meter, the protective effect decreases to 50%. Always wear a mask in public places and maintain a distance of at least 1 meter.
Entity relationship	“Omicron: transmission pathway – airborne”	The Omicron variant can remain suspended in the air as fine aerosols; poorly ventilated indoor environments are high-risk areas. It is recommended to open windows three times a day, for no less than 30 min each time.
Statistical test result	Older adult groups are concerned about vaccine safety	Tip for seniors: Clinical trials show that among people aged 60 years and older receiving the inactivated vaccine, the rate of severe adverse reactions is <0.1%. The most common reactions are injection-site pain (12.3%) and mild fever (5.1%).

This table exemplifies the resource generation for the overarching theme “Preventive and Control Measures” as defined in Table 2. The specific focus here is on the “Personal Protection” sub-topic.

#### 3.2.3 Evaluation results of health education resources

##### 3.2.3.1 TAM model validation and path analysis

Analysis of the 305 valid questionnaire responses indicated a relatively high level of technology acceptance for the health education resources, with an average score of 4.12 out of 5.0. The dimension of Perceived Usefulness (PU) received the highest rating (mean = 4.23). Structural equation modeling confirmed the significance of the hypothesized paths within the Technology Acceptance Model (TAM) framework, as shown in the validation results of the TAM model obtained through TAM model verification and path analysis are shown in Table 4.

**Table 4:** Path coefficients and hypothesis testing results in the technology acceptance model (TAM).

Hypothesized path	Standardized *β*	*t*-value	Conclusion
PEOU → PU	0.38	5.213	Supported
PEOU → BI	0.31	4.027	Supported
PU → BI	0.42	6.872	Supported
eHL × PU → BI	0.18	2.896	Supported
eHL × PEOU → BI	0.22	3.125	Supported

*Path coefficients and hypothesis testing results in TAM:*

1. Perceived Usefulness (PU) exerted the strongest influence on Behavioral Intention to Use (BI) (*β* = 0.42, *p <* 0.001). Users reported that the resources “helped them quickly grasp key protective measures” (mean = 4.31).
2. Perceived Ease of Use (PEOU) significantly impacted both PU (*β* = 0.38, *p <* 0.01) and BI (*β* = 0.31, *p <* 0.01). Among various formats, infographic-based content received the highest score in terms of ease of comprehension (mean = 4.25).
3. The moderating role of eHealth Literacy (eHL) was also significant. Respondents with high eHL placed greater emphasis on the credibility of information sources (*β* = 0.28), while those with low eHL relied more on simplicity of presentation (*β* = 0.51).

##### 3.2.3.2 Validation results of the TAM model

The validation results of the TAM model obtained through TAM model verification and path analysis are shown in Table 4.

##### 3.2.3.3 User preferences and directions for optimization

Chi-square tests revealed statistically significant associations between resource formats and user characteristics:

1. *Age:* Respondents aged 60 years and above showed a strong preference for video/audio formats (*χ*^2^ = 26.8, *p <* 0.001), whereas those aged 18–30 years favored infographic or text-based formats (45.2%).
2. *Educational attainment:* Individuals with less than a high school education were more reliant on community broadcasting as an information source (Odds Ratio = 3.21), while college-educated users primarily preferred social media platforms (78.3%).
3. *eHealth Literacy (eHL):* Participants with high eHL strongly emphasized the need for clearly cited information sources (mean importance rating = 4.35/5.0). Those with low eHL expressed a high demand for dialect-based interpretations (72.6%), indicating the importance of linguistic accessibility.

The results indicated, as shown in Table 5, that trust in official information sources (OR = 2.05) and eHealth literacy levels (OR = 1.87) were key factors contributing to the perceived effectiveness of health education resources (*p <* 0.001). These findings support the relevance of the “source credibility” theory in Library and Information Science, particularly in the context of emergency public health communication.

**Table 5:** Logistic regression analysis of factors influencing the effectiveness of health education resources.

Variable	Odds ratio	Std. err.	z	*P* > |z|
Age60+	0.76	0.12	1.73	0.083
College_edu	1.52	0.21	2.89	0.004
High_eHL	1.87	0.18	4.12	0.000
Official_source	2.05	0.25	5.01	0.000

## 4 Discussion

This study demonstrates, through the construction of a framework integrating library and information science theories with information technology, the rapid transformation path from structured epidemic announcements to personalized health education resources. Our findings not only address the urgent need for precise information dissemination during public health crises but also provide new insights for interdisciplinary research in health communication and LIS. The following discussion will revolve around the study’s theoretical contributions, practical significance, limitations, and future directions.

### 4.1 Practical value of library and information science

The contribution of Library and Information Science (LIS) to the creation of educational health resources is revolutionary. Traditional creation of health education resources often relies on manual work by experts, making the process slow and difficult to customize. In contrast, LIS employs various algorithms and models to transform the creation of health education resources from the traditional approach of “relying on individual expert experience and intuition” to a “data-driven, automated, and quantifiable intelligent process.”

This study confirms the innovative application potential of LIS technologies in public health crisis management (32, 33). The integration of LIS with public health represents an interdisciplinary approach and a novel, effective strategy. The knowledge graph not only enables semantic structuring of bulletin information but also supports inference-based reasoning, which aids in identifying knowledge blind spots—such as the weak semantic association between “aerosol transmission” and “ventilation measures” (34–35). These insights can inform the optimization of health education content.

The resource generation rule base embodies the theory of knowledge recombination in emergency scenarios (36). It transforms fragmented epidemic data into a systematized science communication framework, aligning with the applied teaching philosophy of Henan North Medical College, which advocates for translating professional knowledge into real-world public engagement practices.

The TAM-based evaluation further reveals the moderating role of eHealth literacy, deepening our understanding of the digital divide (37, 38). Individuals with high eHealth literacy prioritize source credibility and information timeliness, while those with lower literacy levels depend more on format accessibility (e.g., dialect interpretation, video formats) (18). This calls for stratified service strategies among information providers—such as offering data traceability tools for high-literacy users, and developing scenario-based videos or catchy rhymes for audiences with lower literacy.

This finding aligns with research conducted in Rizhao City, which showed that 87.5% of residents obtain health information via WeChat or Weibo, but their ability to interpret such information varies significantly across different demographic groups (39, 40).

### 4.2 Optimization pathways for emergency health education

Based on the aforementioned research findings, user acceptance of health education resources is primarily influenced by perceived usefulness, perceived ease of use, eHealth literacy, and trust in official information sources. To translate these empirical findings into practical strategies, we propose the following LIS-driven framework for optimizing emergency health education, aiming to shift health communication from a “broadcast” model to a “targeted dialogue” paradigm.

#### 4.2.1 Tiered resource generation mechanism

This study confirms that users’ eHealth literacy (eHL) and their need for information depth vary significantly (for example, the moderating effect of eHL, OR = 1.87). Therefore, we propose a hierarchical resource generation mechanism to address heterogeneous user needs.

Professional version: Provides open data API and knowledge graph query functions (such as querying “Evolution of COVID-19 Measures in Suzhou”). This meets the high eHL users’ demand for information depth and explorability (supports the discovery of Official_source credibility, OR = 2.05), upgrading the resource from an “information product” to a “knowledge tool.”

Public version: Using a “1 X” content structure, which consists of one core fact sentence paired with multiple extended interpretations (such as charts and examples). This design directly serves users who are sensitive to perceived ease of use (such as low eHL groups) by reducing cognitive load through simplified information architecture, ensuring broad accessibility of basic protection knowledge.

#### 4.2.2 Data-driven multimodal adaptation strategy

The choice of resource format should be informed by user characteristics. Analysis of 305 questionnaires identified age and eHealth literacy as the primary determinants of format preference. Consequently, multimodal adaptation mechanisms must prioritize these core dimensions to dynamically select the most effective resource format for different user groups.

#### 4.2.3 Credibility enhancement design

Regression analysis identified trust in official information sources as one of the strongest predictors of perceived effectiveness (OR = 2.05). Therefore, embedding this trust into resource design is crucial. We propose the following actionable “credibility design” elements:

Source citation: e.g., “Source: National Health Commission bulletin, March 2025.”

Timeliness indicator: e.g., “Last updated: 3 days ago.”

Myth-busting modules: e.g., “Garlic does not kill viruses (Evidence Level A).”

### 4.3 Limitations and future directions

The proposal of this framework is based on the empirical findings of this study, but its development and refinement still face several limitations, which also point the way for future research:

This study has the following limitations:

1. The data source was limited to official government bulletins; future research could incorporate multi-source information, including social media content and expert interviews.
2. The evaluation sample lacked coverage in rural regions, which accounted for only 23.1% of participants.
3. The rule base for resource generation relied heavily on manually predefined rules, which could be further optimized using machine learning techniques.
4. Information related to the epidemic varies to some extent in different stages: Evaluation of Health Education Resources was developed after all three phases of the pandemic. The content related to pandemic preparedness may differ, and relevant information may also vary during any pandemic and at its conclusion.
5. Lack of qualitative feedback: This study overlooked the subtle reactions of the public.
6. Limitations in terms of universality: findings based on Chinese government gazettes may not be fully applicable to the cultural or political contexts of other countries.
7. Automation Transparency: The automatic generation feature can be further improved through more precise documentation or reproducibility.

Future research in Library and Information Science within the public health domain may focus on the following areas:

1. Cross-platform information integration: Development of a comprehensive knowledge graph that fuses data from bulletins, academic literature, and social media.
2. Dynamic user profiling: Implementation of personalized recommendation systems based on real-time tracking of user information behavior.
3. Metaverse applications: Design of virtual health education training environments utilizing immersive technologies.
4. Technology integration: Further explore artificial intelligence and machine learning to enhance the real-time responsiveness of health education tools.
5. Explore the adaptability of this model to health crises in other languages, regions, or types. The model considers administrative hierarchy and risk levels, making it applicable, but it is necessary to investigate multilingual tools and the development of cross-language knowledge graphs and to replace components to complete the modeling.

## 5 Conclusion

Grounded in library and information science theories, this study established a rapid transformation pathway from epidemic announcements to health education resources. Using knowledge graphs and association rule mining, it revealed the relationship between content structure and prevention/control elements, and accordingly designed an automated resource generation mechanism. Empirical research demonstrated that user adoption of these resources is primarily driven by perceived usefulness and ease of use, with e-health literacy playing a significant moderating role. Effective health literacy interventions essentially shift public health communication from “broadcasting” to “targeted dialogue.”

This interdisciplinary framework integrating LIS and public health has significant practical application potential. During public health emergencies, it can be directly deployed within local health departments’ emergency response processes, enabling real-time conversion and precise delivery of personalized educational materials from authoritative announcements. To promote implementation and dissemination, the following specific steps are recommended: first, collaborate with local Centers for Disease Control and Prevention (CDCs) to conduct regional pilot applications; second, integrate this knowledge development framework into provincial and municipal public health emergency response plans; third, establish multi-stakeholder collaboration involving public health officials, community workers, and public representatives.

Research results indicate that the integration of LIS with public health represents an effective interdisciplinary strategy. The associated tools not only improve the efficiency of health education resource development but also support precision communication through user-centered demand analysis. The knowledge development framework proposed in this study provides a methodological foundation for emergency health education, and its analysis–generation–evaluation closed-loop model can be extended to other crisis contexts. This contributes to the broader goal of building a resilient information society through LIS-driven solutions.

## Data Availability

The datasets presented in this study can be found in online repositories. The names of the repository/repositories and accession number(s) can be found in the article/supplementary material.
