# Supplementary material for: Research on rapid construction methods and evaluation of health education resources in public health emergencies based on knowledge development
Source: Front Public Health. 2025 Dec 17;13:1686843. doi: 10.3389/fpubh.2025.1686843 (PMC12753991; doi:10.3389/fpubh.2025.1686843)
Supplement: Supplementary file 1 [file Data_Sheet_1.PDF]

2.3.3 Multimodal Adaptation Mechanism

The multimodal adaptive mechanism plays a core “generator” role in the “analyze-generate-evaluate” framework. [31]It inherits the output from knowledge analysis and directly drives the concrete actions of resource generation, serving as an intelligent bridge between “what the content is”and “how the form should be” .This mechanism is implemented through a conditional selection function, with the user profile as input and the recommended multimodal resource format as output. The internal rules of the function are designed based on prior research and theoretical inferences about the information processing characteristics of different user groups.

Resource formats were adapted to user characteristics through a conditional selection mechanism:

```python

def select_modality(user_profile):

#Older adults

if user_profile["age"] >= 60:

return "Audio narration + large-font text"

#Education level below high school

elif user_profile.get("education_level") in ["Below high school"]:

return "Short video + infographic"

else:

return "Interactive Q&A + long-form text"

```

4.2.2 Data-Driven Multimodal Adaptation Strategy

The choice of resource format should be based on insights into user characteristics. Analysis of 305 questionnaires revealed the key factors influencing preferences for resource formats, with age and eHealth literacy being the main determinants in selecting resource formats.

```mermaid
```

pie

title Basis for Resource Format Selection

“Age Factor” : 42

“eHealth Literacy” : 33

“Education Level” : 19

“Regional Culture” : 6

```

This empirical finding requires that multimodal adaptation mechanisms must prioritize core dimensions such as age and eHL, dynamically selecting the most effective resource format for different groups.
```
