# Supplementary material for: Research on rapid construction methods and evaluation of health education resources in public health emergencies based on knowledge development
Source: Front Public Health. 2025 Dec 17;13:1686843. doi: 10.3389/fpubh.2025.1686843 (PMC12753991; doi:10.3389/fpubh.2025.1686843)
Supplement: Supplementary file 2 [file Table_1.DOCX]

**Appendix A: TransH Code Review**

```python

# TransH Knowledge Graph Training

import torch

import torch.nn as nn

import torch.nn.functional as F

class TransH(nn.Module):

  def __init__(self, ent_size, rel_size, dim):

    super(TransH, self).__init__()

    self.ent_emb = nn.Embedding(ent_size, dim)

    self.rel_emb = nn.Embedding(rel_size, dim)

    self.norm_emb = nn.Embedding(rel_size, dim)  # Projection plane normal vector

  def forward(self, h, r, t):

    h_emb = self.ent_emb(h)

    r_emb = self.rel_emb(r)

    t_emb = self.ent_emb(t)

    w_r = F.normalize(self.norm_emb(r), p=2, dim=1)  # Normalize normal vector

    # Project to the relation plane

    h_proj = h_emb - torch.sum(h_emb * w_r, dim=1, keepdim=True) * w_r

    t_proj = t_emb - torch.sum(t_emb * w_r, dim=1, keepdim=True) * w_r

    # Calculate translation distance

    return torch.norm(h_proj + r_emb - t_proj, p=2, dim=1)

```

**Appendix B: Decision Tree Rule Set Review**

| **Rule ID** | **Condition** | **Output** |
| --- | --- | --- |
| 1 | Village clinic visit rate ≥ 33.5% AND group activity frequency ≥ 2 times/week | Red (High Risk) |
| 2 | Village clinic visit rate ≥ 33.5% AND group activity frequency < 2 times/week | Yellow (Medium Risk) |
| 3 | Village clinic visit rate < 33.5% AND proportion of elderly population > 40% | Yellow (Medium Risk) |
| 4 | Village clinic visit rate < 33.5% AND proportion of elderly population ≤ 40% | Green (Low Risk) |
