# Supplementary material for: Research on rapid construction methods and evaluation of health education resources in public health emergencies based on knowledge development
Source: Front Public Health. 2025 Dec 17;13:1686843. doi: 10.3389/fpubh.2025.1686843 (PMC12753991; doi:10.3389/fpubh.2025.1686843)
Supplement: Supplementary file 3 [file Table_2.DOCX]

Here is a comprehensive list of additional generation rules, structured for a supplemental file, that expand upon the examples given. These rules cover various dimensions like user profile, content complexity, and delivery platform.

***

### **Supplemental File S1: Comprehensive Rule Set for Health Education Resource Generation**

This document details the complete set of rules used by the Intelligent Generation System for Health Education Resources. These rules are applied by the **Resource Type Selector** and **Content Template Matching** modules after the **Knowledge Analysis Module** processes the original text and user profile.

#### **1. Rules Based on User Age**

| Rule ID | Condition | Action | Rationale |

| :--- | :--- | :--- | :--- |

| **AGE-01** | `User Age ≥ 60` | Adopt a "**Video + Audio Narration**" format by default. Use large, high-contrast fonts in any text overlays. | Reduces cognitive load, accommodates potential visual impairment, and leverages a familiar media format. |

| **AGE-02** | `User Age ≤ 25` | Prioritize "**Interactive Infographic**" or "**Short-Form Video (< 60s)**" formats. Allow for social sharing. | Aligns with media consumption habits of younger demographics, promoting higher engagement. |

| **AGE-03** | `35 ≤ User Age ≤ 55` | Default to "**Detailed Article with Bullet Points**" or "**Comprehensive FAQ Sheet**". | Caters to an audience that often seeks in-depth, credible information for personal or family decision-making. |

#### **2. Rules Based on Detected Medical Entities & Concepts**

| Rule ID | Condition | Action | Rationale |

| :--- | :--- | :--- | :--- |

| **ENT-01** | Entity `"Omicron"` is recognized. | Link to a dedicated, updated science communication page on **Virus Variants**. | Provides context and directs users to a centralized, authoritative source for evolving information. |

| **ENT-02** | Entity `"Myocarditis"` OR `"Pericarditis"` is recognized. | Insert a **Comparative Risk Chart** (e.g., risk from infection vs. risk from vaccination). | Addresses a common concern with clear, data-driven context to mitigate misinformation. |

| **ENT-03** | Entity `"Booster Dose"` is recognized AND `User Age` is present. | Dynamically insert text: "**You are in the [User Age] age group. Current guidelines recommend...**" and pull the latest guideline. | Personalizes recommendations, increasing relevance and the likelihood of adherence. |

| **ENT-04** | Entity `"Mask"` is recognized. | Insert a **Step-by-Step Visual Guide** on proper mask wearing and disposal. | Correct knowledge gaps in the correct usage of a primary protective measure. |

#### **3. Rules Based on Association Rules (Mined Relationships)**

| Rule ID | Condition | Action | Rationale |

| :--- | :--- | :--- | :--- |

| **ASSOC-01** | Association Rule `"Vaccine Efficacy" → "Side Effects"` is detected with high confidence. | Insert a **Side-Effect Prevalence Table** adjacent to the efficacy data. Use clear headings like "Very Common (>10%)", "Rare (<0.1%)". | Proactively addresses the most common user query pattern, building trust through transparency. |

| **ASSOC-02** | Association Rule `"Paxlovid" → "Drug Interaction"` is detected. | Insert a prominent **"Check for Drug Interactions"** warning box and link to an official drug interaction checker. | Highlights critical safety information that requires professional consultation. |

| **ASSOC-03** | Association Rule `"Long COVID" → "Fatigue"` is detected. | Link to a dedicated resource on **"Managing Post-COVID Symptoms"** that includes practical advice from physiotherapists. | Provides actionable support for a prevalent and distressing condition. |

#### **4. Rules Based on User's Education Level (from Profile)**

| Rule ID | Condition | Action | Rationale |

| :--- | :--- | :--- | :--- |

| **EDU-01** | `Education Level = "High School or Below"` | Use analogies and simple language. Avoid complex statistical terms. Use **Icons and Pictograms** heavily. | Ensures comprehension for audiences with lower health literacy. |

| **EDU-02** | `Education Level = "University or Above"` | May include more technical data, **Forest Plots** from studies, or discuss **Confidence Intervals**. The format can be a detailed report. | Meets the expected depth of information for a more academically inclined audience. |

#### **5. Rules Based on Platform & Accessibility**

| Rule ID | Condition | Action | Rationale |

| :--- | :--- | :--- | :--- |

| **PLAT-01** | Resource is to be delivered via `SMS` or `Text-Heavy Social Media (e.g., X/Twitter)`. | Generate a **Concise Text Summary** (< 280 characters) with a link to the full resource. | Optimizes for platform constraints and user behavior. |

| **PLAT-02** | `User Accessibility Requirement = "Screen Reader"` | Ensure all images have **Descriptive Alt Text**. Prioritize a well-structured HTML page over a PDF. | Guarantees access for visually impaired users. |

| **PLAT-03** | `User Language ≠ English` | Trigger the **Localization Module** to generate resources in the user's preferred language, using culturally appropriate examples. | Ensures understanding and relevance across diverse populations. |

This expanded rule set demonstrates how the system can move from a simple prototype to a robust and reproducible framework for generating personalized, effective health education materials.
